# Clinical application of the REVEAL™ fluorescence loupe in oral lesion assessment: a five-case clinical note

**DOI:** 10.1007/s10103-026-04861-0

**Published:** 2026-03-23

**Authors:** Matheus Albino Souza, Letícia Copatti Dogenski, Gabriele Chitolina, Eduarda Lunelli Ferrari, Gisele Rovani, Liviu Steier, José Antônio Poli de Figueiredo, João Paulo De Carli

**Affiliations:** 1 Universidade de Passo Fundo, Passo Fundo, Brazil; 2 University of Pennsylvania, Philadelphia, USA; 3 Federal University of Rio Grande do Sul, Porto Alegre, Brazil

**Keywords:** Autofluorescence, Oral lesions, Diagnosis, Optical imaging

## Abstract

**Purpose:**

To illustrate the clinical use and feasibility of the REVEAL™ fluorescence loupe as an adjunctive tool in the chairside assessment of inflammatory, potentially malignant, and malignant oral lesions.

**Methods:**

Five patients with oral lesions were clinically examined under natural light and with the REVEAL™ loupe. When indicated, biopsy and histopathological analysis were performed

**Results:**

Inflammatory lesions exhibited a bright and homogeneous red autofluorescence pattern. Actinic cheilitis demonstrated heterogeneous brownish autofluorescence with whitish areas. Squamous cell carcinomas did not show significant autofluorescence contrast. The presence of biofilm was highlighted by a distinct orange signal.

**Conclusion:**

The REVEAL™ loupe may support the clinical assessment of oral lesions by illustrating autofluorescence patterns associated with underlying tissue changes. However, its use should be considered complementary to biopsy, not a diagnostic substitute.

## Introduction

Although biopsy and histopathological examination remain the gold standard for diagnosing various lesions in the oral cavity, new methods to assess the potential presence and degree of epithelial dysplasia have become increasingly important in recent clinical studies. In this context, optical autofluorescence imaging has gained prominence by offering a non-invasive, painless, and rapid method to collect real-time information and acquire broad-spectrum images of oral mucosal alterations. Therefore, optical autofluorescence is a technique that can support the detection of such alterations, enabling early diagnosis with a greater likelihood of favorable prognosis, in addition to guiding the boundaries for potential biopsies to confirm diagnosis [1, 2].

Within this context, the REVEAL™ device (Designs for Vision Inc., Bohemia, USA) stands out as a portable, wearable apparatus consisting of magnifying loupes with filters and an external light source. The emitted light spectrum, when directed intraorally, has the potential to produce photoluminescence (emission) due to the autofluorescent properties of oral structures [3]. The wavelengths emitted by the light source stimulate the observed tissues, whose endogenous fluorophores emit colors resulting from the difference between the absorption and emission wavelengths. Despite providing high-resolution imaging, further studies on REVEAL™ are needed to confirm its effectiveness [4] and characterize the appearance of different oral mucosal changes under its light.

Given this background, the objective of the present study is to descriptively report the clinical application and observed autofluorescence patterns obtained with the REVEAL™ loupe in a small illustrative case series, without evaluating diagnostic accuracy.

### Case description

This case series report was approved by a Research Ethics Committee (nº7.250.422).

### Sample selection

Patients treated at a Stomatology Outpatient Clinic (AE) of a School of Dentistry (CO) were selected. These individuals sought care for the evaluation and management of oral mucosal alterations. After screening and initial extraoral and intraoral clinical examination, patients with a probable diagnosis of inflammatory lesions (lichen planus and traumatic ulcer), potentially malignant lesions (leukoplakia and actinic cheilitis), and malignant lesions (squamous cell carcinoma, SCC) of the oral cavity and/or the vermilion border of the lips were invited to take part in the study after reading and signing a specific informed consent form.

This was a convenience sample of illustrative cases selected to represent different clinical categories (inflammatory, potentially malignant, and malignant lesions). The cases were not consecutively recruited for research purposes. Other lesions evaluated during the same period were not included, as the aim was to provide a didactic clinical spectrum rather than an epidemiological representation.

### Inclusion and exclusion criteria

Patients presenting with at least one lesion showing clinical characteristics of lichen planus, traumatic ulcer, leukoplakia, actinic cheilitis, or squamous cell carcinoma, regardless of the lesion’s location, were included in the study. Lesions with features suggestive of malignant transformation or malignancy were all referred for incisional biopsy and histopathological examination to confirm the clinical diagnosis. To be included, patients were required to agree to the terms of the informed consent form and allow collection of personal data (age, gender, occupation, medication use, tobacco and/or alcohol consumption). Patients with lesions that did not exhibit characteristics of inflammatory, potentially malignant, or malignant lesions, such as those caused by microorganisms (e.g., candidiasis or other infections), were excluded from the study.

### Characterization of oral lesions

**Fig. 1:**
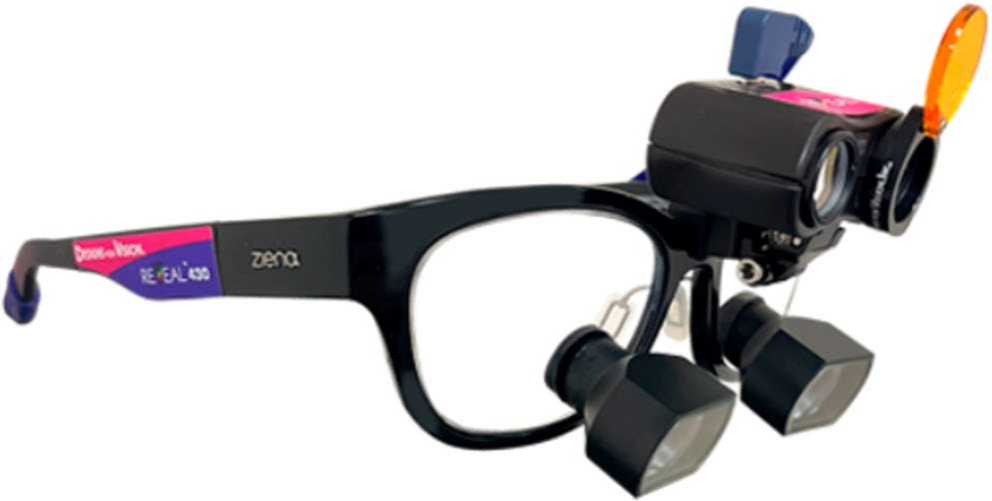
REVEAL™ device. Source: Designs for Vision Inc., Bohemia, USA

With the patient seated in the dental chair, photographs of the lesions in the oral cavity and/or vermilion border of the lip were taken using a smartphone (Apple iPhone 15, Apple Inc., Cupertino, CA, USA), both under natural light and using the REVEAL™ device (Fig. 1).

The REVEAL™ device is equipped with a blue-violet LED excitation light (approximately 405–450 nm, according to manufacturer specifications) and has an emission filter integrated into the loupe system. Examinations were conducted in a dental operatory with overhead light turned off to minimize ambient light interference. The working distance ranged from approximately 30–40 cm, allowing optimal focus through the magnification system. Patients used special protective eyewear provided by the manufacturer during short-term intraoral examination. No adverse effects were reported during use of the loup.

Smartphone camera settings were maintained under automatic exposure mode; white balance was not manually adjusted. Images were subsequently standardized for brightness and contrast only, without altering color hue.

autofluorescence interpretation was performed descriptively by two calibrated clinicians. Observers were not blinded to the clinical presentation, as autofluorescence assessment was integrated into the routine clinical examination. No inter-examiner agreement analysis was performed.

To obtain the autofluorescence images, the device was positioned at a distance that allowed optimal focus through the loupe attached to the visor. The device was held in place by one operator, while another aligned the smartphone camera directly with the loupe lens, so that the images captured with the REVEAL™ device reflected the dentist’s actual field of view (fluorescence light combined with the magnification lens).

autofluorescence was evaluated based on: (1) intensity (increased, reduced, absent), (2) coloration (red, brown, orange, normochromatic), and (3) margin definition (well-defined or diffuse). No semi-quantitative autofluorescence intensity measurements were performed. The interpretation of autofluorescence patterns was descriptive.

### Incisional biopsy and histopathological analysis

When necessary, an incisional biopsy of the most representative area of the oral lesion was performed by an experienced oral pathologist. The biopsy specimens were sent to an Oral Pathology Laboratory for histopathological examination, carried out by a qualified oral pathologist, to confirm the clinical diagnosis. The researchers involved in this study did not perform the biopsy procedures but had access to the histopathological diagnosis and epithelial dysplasia grading, when present, as they followed up with the patients during their return visits and delivery of the histopathology reports.

Each patient included in the study received the most appropriate treatment based on their clinical and histopathological diagnoses, in accordance with the guidelines and protocols of the CO. Treatments were conducted at the AE clinic and/or referred to a hospital facility when specialized medical care was required. This study did not delay or interfere with the biopsy or treatment process; rather, it introduced an auxiliary observational tool to the routine diagnostic workflow.

## Results

### Case 1

**Fig. 2:**
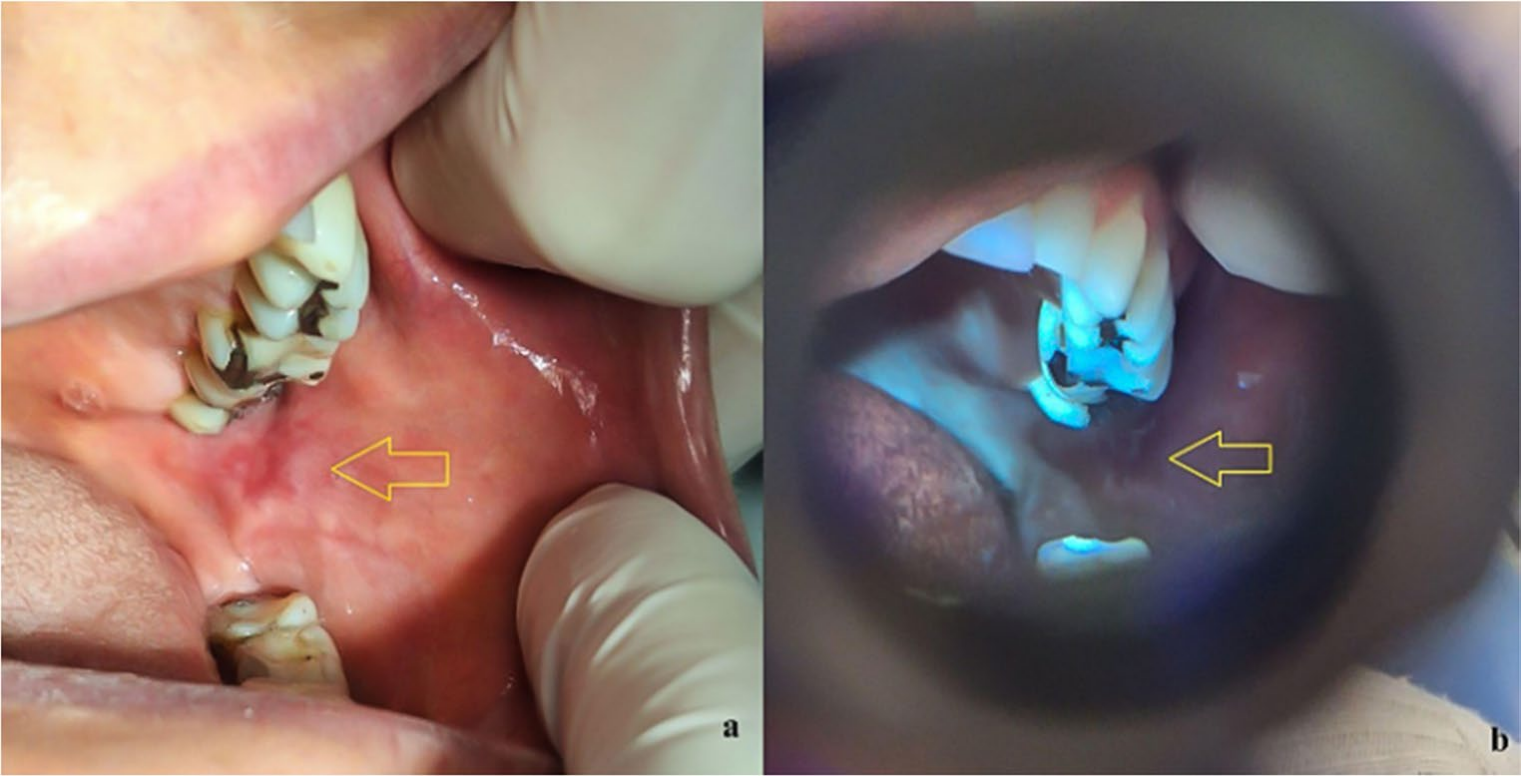
Fig. 2a Slightly eroded aspect of the left buccal mucosa, with a clinical diagnosis of erosive lichen planus (clinical appearance under white light) Fig. 2b Corresponding autofluorescence image obtained with REVEAL™ loupe under standardized conditions, showing a distinct, well-defined bright red coloration, in contrast with the adjacent buccal mucosa. Autofluorescence images were standardized for brightness and contrast adjustment only, without altering color hue

A 66-year-old retired female patient, with no history of smoking or alcohol consumption, sought care at the AE clinic reporting painful ulcerated lesions on the bilateral buccal mucosa, left lateral tongue border, and tongue apex. Clinical examination revealed superficial ulcers with flat borders, a reddish base, and an erythematous halo on both the right and left buccal mucosa. On the left tongue border and apex, slight mucosal erosion with a reddish appearance was observed (Fig. 2a). The patient reported being under significant emotional stress. The probable clinical diagnosis was erosive lichen planus.

Under fluorescence light from the REVEAL™ loupe, the ulcerated lesions showed a distinct, well-defined bright red coloration in contrast with the adjacent buccal mucosa, which appeared normochromatic (Fig. 2b).

The patient received local application of 3 joules (J) of infrared laser, applied directly to the painful areas on the buccal mucosa and tongue, continuing until pain symptoms resolved. Nystatin mouth rinses were also prescribed to treat a suspected associated fungal infection. The patient was informed that the lesion, although autoimmune in nature, could be exacerbated by stress. At the follow-up appointment after 7 days, a reduction in the erosive and reddish appearance of the lesions was noted, as illustrated by the image of the patient’s left buccal mucosa. The patient also reported improvement in pain symptoms.

### Case 2

**Fig. 3:**
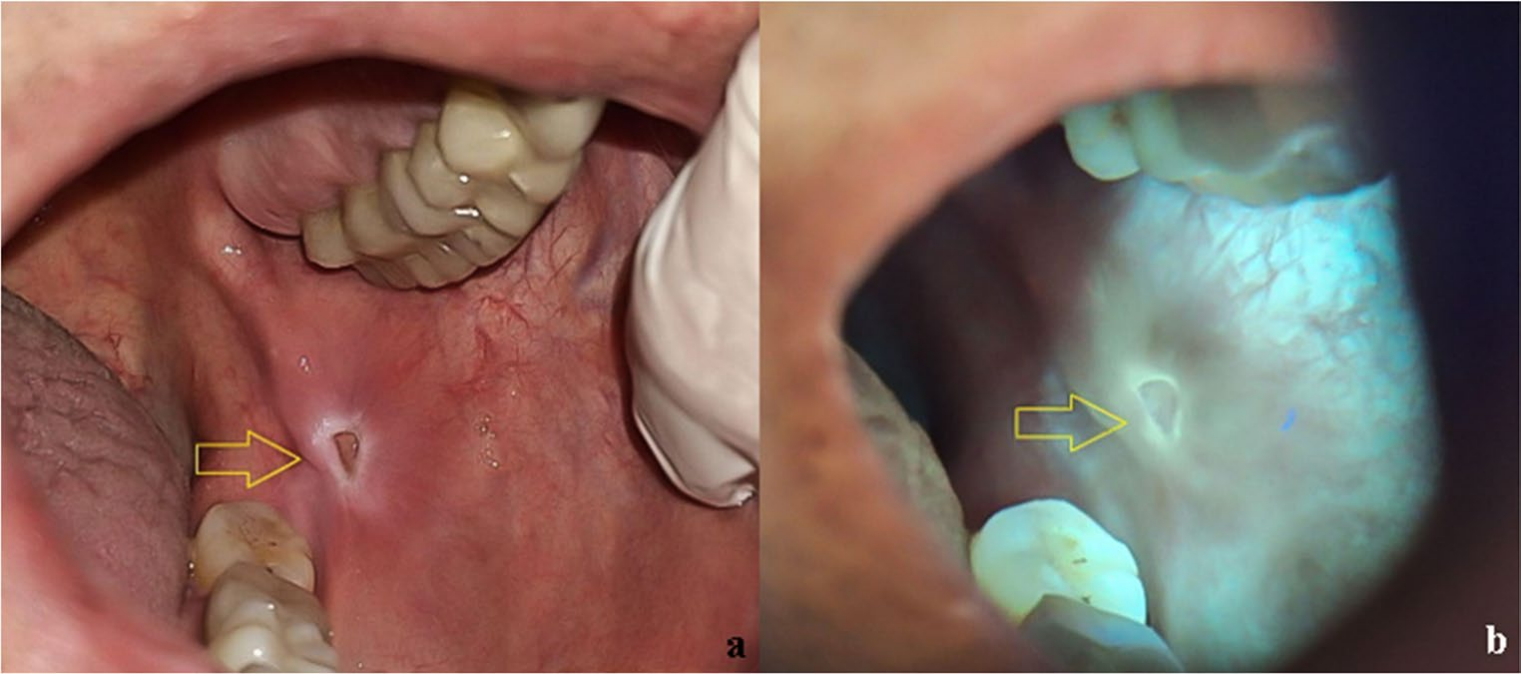
Fig. 3a An ulcer with a depressed yellowish center, shallow and leukoplastic borders on the left buccal mucosa (clinical appearance under white light) Fig. 3b Corresponding autofluorescence image obtained with REVEAL™ loupe under standardized conditions, showing a distinct bright red coloration diffusing toward the normochromic adjacent epithelium. Autofluorescence images were standardized for brightness and contrast adjustment only, without altering color hue

A 78-year-old retired male patient sought care at the AE reporting the presence of an ulcer with a depressed and yellowish center, shallow and leukoplastic borders, measuring 3 × 5 mm (Fig. 3a). The lesion had been noticed on the left buccal mucosa for at least one month, following direct trauma from a sleep apnea control device.

Under the fluorescence light of the loupe, the ulcerated lesion showed a distinct bright red coloration that diffused toward the adjacent epithelium, which appeared normochromic (Fig. 3b).

With clinical diagnostic hypotheses of traumatic ulcer and leukoplakia, an excisional biopsy was performed. Histopathological analysis revealed a squamous epithelium with a centrally focal ulcerated area covered by a dense chronic inflammatory infiltrate predominantly composed of phagocytes (macrophages and neutrophils) and platelet fibrin. The adjacent epithelium was hyperplastic with hyperparakeratosis and also showed a chronic underlying infiltrate with a predominance of lymphocytes, plasma cells, and neocapillaries, resembling granulation tissue. In other fields, the epithelium presented as hyperplastic, acanthotic, or bulbous. Across the entire specimen, hyperparakeratosis was present, and no inflammatory infiltrate was observed in the chorion of these adjacent epithelia.

Thus, the final diagnosis was a nonspecific ulcer and, adjacent to it, acanthosis and hyperparakeratosis. The patient underwent polishing of the sharp edges of the posterior teeth and the sleep apnea control device to prevent trauma that could lead to recurrence of the lesion.

### Case 3

**Fig. 4:**
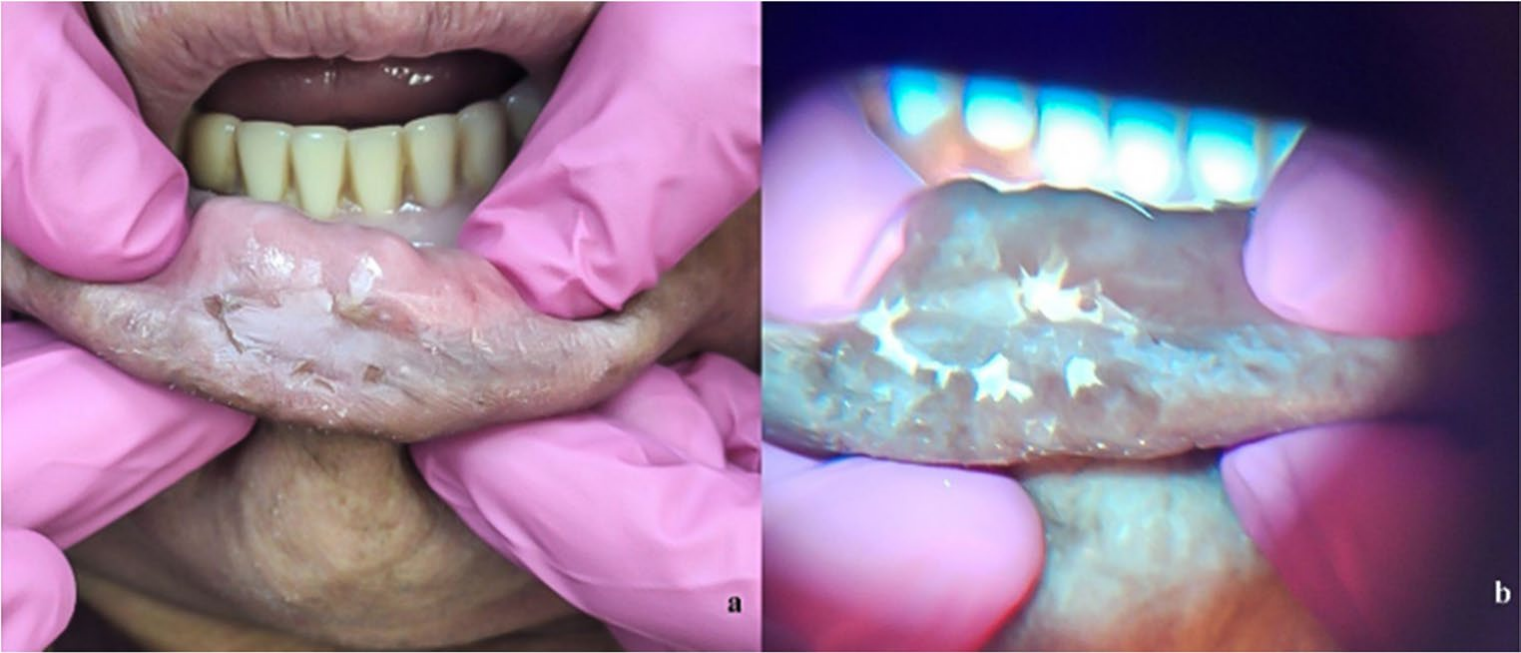
Fig. 4a A lesion on the lower lip, showing an ulcer and a leukoplastic plaque with a rough surface (clinical appearance under white light) Fig. 4b Corresponding autofluorescence image obtained with REVEAL™ loupe under standardized conditions, with chestnut brown tones throughout the lesion and a mixture of whitish tones in some areas. Autofluorescence images were standardized for brightness and contrast adjustment only, without altering color hue

A 70-year-old male patient, farmer, with a history of excessive sun exposure and use of agricultural pesticides without protection. He did not report smoking or alcohol habits. He sought care at AE complaining of an ulcer on the lower lip vermilion, which had appeared three months earlier. On clinical examination, an ulcer and a leukoplastic plaque with a rough appearance, measuring 10 × 20 mm, were observed (Fig. 4a). The patient reported having used lip balm for 15 days, during which no clinical changes in the lesion were noted. Given the clinical diagnosis of actinic cheilitis, an excisional biopsy was performed to remove the affected soft tissue.

Under the fluorescence loupe light, the lip lesion appeared brown throughout its entire extent, with shades of chestnut brown. In some areas of the lesion, a mixture of whitish tones was observed (Fig. 4b).

Histopathological examination showed squamous epithelium with a basal layer that was sometimes bulbous and sometimes atrophic and acanthotic, a thick granular layer, and superficial hyperorthokeratosis. In the chorion, chronic inflammatory infiltrate and solar elastosis were present. Thus, a diagnosis of mild epithelial dysplasia was made, and the patient was instructed on necessary care to avoid further oral and/or lip vermilion lesions and advised to return for dental follow-up every six months.

### Case 4

**Fig. 5:**
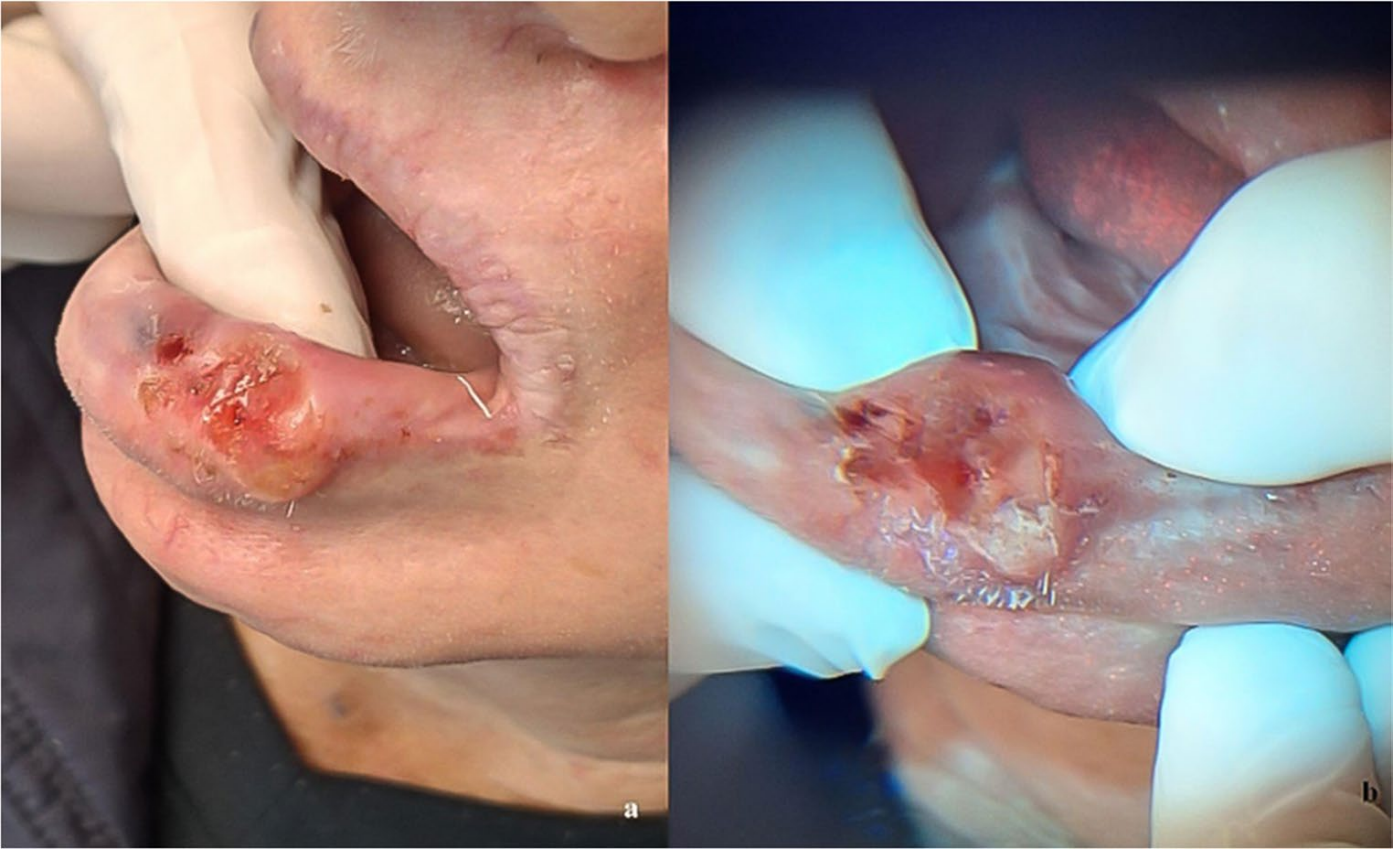
Fig. 5a Aspect of the ulcer with raised borders and depressed center on the left lower lip (clinical appearance under white light) Fig. 5b Corresponding autofluorescence image obtained with REVEAL™ loupe under standardized conditions, identical to that observed under natural light, with no color changes. Autofluorescence images were standardized for brightness and contrast adjustment only, without altering color hue

Male patient, 64 years old, farmer, with a history of excessive sun exposure and contact with agricultural pesticides without protection. He did not report smoking or alcohol use. He sought care at the AE reporting the presence of a painless lesion on the left lower lip. On clinical examination, a 2 × 2 cm ulcer with raised edges and a depressed center was observed, which appeared 3 months ago and progressed rapidly (Fig. 5a). Under the loupe light, the lip lesion appeared identical to that observed under natural light, with no color changes (Fig. 5b).

Given the probable diagnosis of SCC, an incisional biopsy was performed to remove two soft tissue fragments. Histopathological examination showed dysplastic growth of the squamous epithelium invading the connective tissue, forming epithelial islands with keratin pearls, and cells with pleomorphic nuclei and atypical mitoses. In the connective tissue, there was a chronic inflammatory infiltrate. No epithelial embolization was found in blood vessels or nerve bundles in the specimen. Thus, a diagnosis of well-differentiated SCC (grade I) was confirmed, and the patient was referred to a specialized service to initiate treatment.

### Case 5

**Fig. 6:**
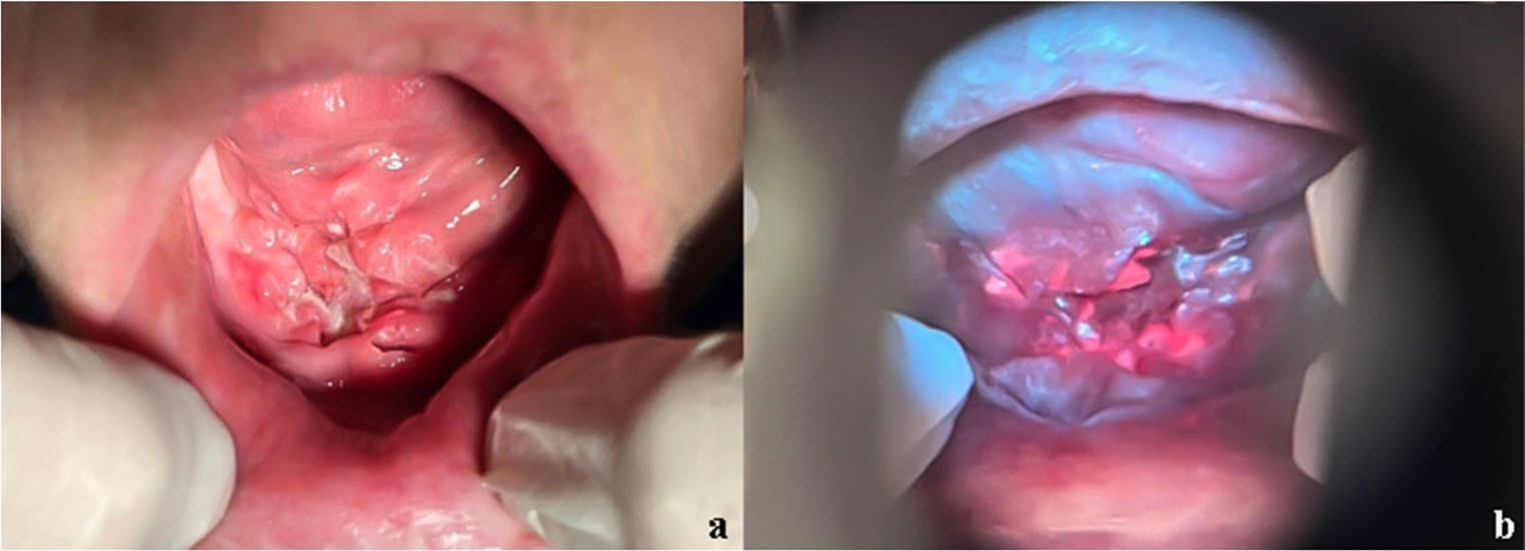
Fig. 6a An ulcer with a necrotic and depressed center, red and covered by biofilm on the entire surface, with elevated edges and a moriform appearance (clinical appearance under white light) Fig. 6b Corresponding autofluorescence image obtained with REVEAL™ loupe under standardized conditions, with the oral tissue appearing identical to that observed under natural light, showing areas of intense orange coloration characteristic of biofilm presence at the site. Autofluorescence images were standardized for brightness and contrast adjustment only, without altering color hue

A 54-year-old female patient, retired, reporting smoking one pack of cigarettes per day for 40 years. She sought care at the AE reporting the presence of a lesion in the anterior region of the floor of the mouth, along the midline. Clinical examination revealed an ulcer with a necrotic and depressed center, red and covered by biofilm on the entire surface, elevated edges with a moriform appearance, approximately 3 × 3 cm in size, hardened to palpation and with spontaneous painful symptoms (Fig. 6a). The presence of a hardened, fixed, and tender submental lymph node was also noted on palpation. The patient reported that the lesion had appeared 4 months earlier and showed progressive growth.

Under the loupe light, the oral tissue appeared identical to that observed under natural light, without changes in coloration. However, a strong orange coloration was observed over the lesion, characteristic of the presence of biofilm at the site (Fig. 6b).

Given the probable diagnosis of SCC, an incisional biopsy was performed to remove three fragments of soft tissue. Histopathological examination showed proliferation of the squamous epithelial covering tissue into the connective tissue in large islands, with cells showing marked pleomorphism, mostly atypical, with giant and hyperchromatic nuclei, others with polygonal nuclei and scant cytoplasm, and numerous bizarre mitoses. In the connective tissue, there was intense chronic inflammatory infiltrate with phagocytes, and epithelial embolization was found in the lumen of capillaries. No nerve bundles were found in the specimen. Thus, the diagnosis of moderately differentiated SCC (grade II) was concluded, and the patient was referred to a specialized service to begin treatment.

### Integrated observation of the cases

The five selected cases represent a progressive spectrum of inflammatory, potentially malignant, and malignant alterations, allowing us to observe how different degrees of tissue involvement influence the autofluorescence patterns visualized with the REVEAL™ device. This heterogeneous selection aims to demonstrate both the potential and the limitations of the device in real clinical scenarios. Table 1 presents the main characteristics of each case, as well as the strengths and limitations of the autofluorescence patterns observed in each of them.

**Table 1:** Summary of clinical presentation, histopathological findings, and observed autofluorescence patterns in the five cases

Case nº	Clinical aspect	Histopathological aspect	Autofluorescence pattern	Strengths	Limitations
1	Erosive lichen planus	No biopsy performed	Bright, well-demarcated red autofluorescence	Allowed differentiation between inflamed areas and normal tissue	Lack of specificity in distinguishing between different inflammatory lesions
2	Traumatic ulcer	Nonspecific ulcer with inflammation	Diffuse bright red autofluorescence	Differentiated the ulcerated area from the adjacent epithelium	Subtle difference between trauma and inflammation
3	Actinic cheilitis	Mild dysplasia	Brown/whitish tones	Pattern distinct from inflammatory lesions	Does not allow prediction of dysplasia grade
4	SCC	Well-differentiated SCC	No contrast	Demonstrated the limitation of the technique in neoplasms	Necrosis and structural loss impair autofluorescence
5	SCC	Moderately differentiated SCC	No contrast + strong orange signal (biofilm)	Biofilm detection, complementary aid	Autoautofluorescence did not contribute to diagnosis

## Discussion

This report presents preliminary clinical observations and does not aim to evaluate diagnostic accuracy. The primary contribution of this clinical note is to illustrate how autofluorescence patterns may help guide the clinical reasoning process during oral lesion assessment. This descriptive case series does not assess sensitivity, specificity, or diagnostic performance.

In the first two cases, the presence of ulcers with clinical features compatible with inflammatory processes was observed. Notably, both lesions exhibited a vivid red coloration under the REVEAL™ loupe light, sharply demarcated from the adjacent mucosa, which remained normochromic. These observations are consistent with patterns commonly reported in inflammatory lesions, in which increased vascularization and erythema correspond to enhanced autofluorescence contrast [5]. This pattern may assist in distinguishing inflammatory processes; however, its diagnostic value requires further validation.

Gorpas et al. [6], in their study aiming to explore the use of time-resolved fluorescence spectroscopy (TRFS) to detect alterations in fluorescence lifetime between oral lichen planus lesions and normal perilesional mucosa, found higher wavelengths in lichen planus lesions compared to normal mucosa. They discuss that this differentiation may be attributed to the reduced presence of collagen and elastin fibers in the affected tissue. The excitation light penetration depth (~ 300 μm) in lichen planus-affected tissue was largely or exclusively limited to the epithelium, whereas in normal perilesional tissue, this depth extended into the lamina propria. The rich collagen and elastin composition of the lamina propria results in stronger fluorescence signals.

In the third case, where an extensive and rough leukoplakic plaque was observed, clinically compatible with a potentially malignant lesion, the autofluorescence revealed a brownish hue with interspersed whitish areas. This pattern differed from that observed in the inflammatory cases, indicating a change in autofluorescence behavior that has been previously reported in dysplastic tissue. The brown areas have been associated in the literature with stromal collagen alterations and increased cellular turnover [7, 8]; however, such biological correlations were not directly measured in this study and remain speculative.

Moreover, studies have shown that dysplastic lesions exhibit increased mitochondrial autofluorescence, attributed to elevated NADH (reduced nicotinamide adenine dinucleotide) levels in epithelial cells [9, 10]. NADH is a fluorescent coenzyme essential for cellular metabolism, particularly in mitochondrial energy production [11]. In normal tissues, its autofluorescence is generally weak and restricted to the basal epithelial layers. However, during epithelial dysplasia, there is an increase in cellular metabolic activity, resulting in elevated NADH levels and enhanced epithelial autofluorescence [12]. These mechanisms have been proposed in the literature and may partially explain the observed patterns.

On the other hand, the last two cases, which presented classic clinical features of malignant lesions, did not demonstrate significant color changes under the REVEAL™ loupe when compared to white light. This lack of contrast may be related to intense tissue structure loss, presence of necrosis, and thick biofilm - factors that negatively interfere with autofluorescence emission and detection [13]. In this regard, Lane et al. [14], in their study aiming to understand how optical properties are altered during oral carcinogenesis, observed that oral tissue autofluorescence varies according to anatomical site and pathological diagnosis. They found that stromal autofluorescence, mainly associated with collagen, significantly decreases in neoplastic lesions.

In one of the cases with classic clinical features of malignant lesions, an orange coloration over the lesion was observed, consistent with biofilm accumulation, a finding supported by the literature which describes this hue as characteristic of bacterial activity [15]. While the absence of autofluorescence limits lesion characterization, the detection of distinct patterns (e.g., orange signal over biofilm) may contribute supplementary visual information during examination.

Overall, the findings of this study corroborate previous reports that tissue autofluorescence can be altered due to morphological, biochemical, and structural changes, such as those present in inflammations, dysplasias, and carcinomas. Although the small sample size limits generalizations, the results indicate the clinical utility of the REVEAL™ loupe as an auxiliary tool in intraoral examination, contributing to the screening and monitoring of oral lesions.

The absence of a distinct autofluorescence pattern in cases of advanced dysplasia and carcinoma highlights an important limitation of the technique, particularly when there is significant disruption of tissue architecture. In such situations, epithelial disorganization, stromal degradation, necrosis, and biofilm accumulation may reduce or mask the intrinsic autofluorescence signal, complicating interpretation.

The primary limitation of this study is the small sample size (*n* = 5), which does not allow generalization of the observations or the calculation of diagnostic performance. In addition, autofluorescence assessment was qualitative rather than quantitative, which limits the reproducibility of the findings. Therefore, the results presented here should be interpreted as preliminary observations, illustrating the potential clinical applicability of the REVEAL™ loupe rather than establishing diagnostic criteria. Additionally, one inflammatory case (erosive lichen planus) did not undergo histopathological confirmation, as the clinical presentation was considered typical and responded to conservative management. This represents a limitation regarding diagnostic confirmation.

Accordingly, the REVEAL™ device should be understood as a complementary tool to clinical examination and histopathological analysis, and not as a substitute for biopsy. Future research with larger samples, standardized imaging protocols, and semi-quantitative or quantitative autofluorescence evaluation is necessary to validate these findings and clarify the diagnostic utility of autofluorescence imaging in oral lesion assessment.

## Data Availability

No datasets were generated or analysed during the current study.

## References

[1] Shavlokhova V, Flechtenmacher C, Sandhu S et al (2021) Feasibility and implementation of ex vivo fluorescence confocal microscopy for diagnosis of oral leukoplakia: preliminary study. Diagnostics (Basel) 11(6):951. 10.3390/diagnostics11060951 34073373 10.3390/diagnostics11060951 PMC8228631

[2] Ribeiro CL, Meireles MR, De Souza Queiroz CD et al (2022) Uso da fluorescência óptica na avaliação da saúde oral de pacientes de uma clínica escola. Rev Fac Odontol Lins 32(1–2):13–20. 10.15600/2238-1236/fol.v32n1-2p13-20

[3] Steier L (2020) Reveal: fluorescence enhanced theragnosis by Designs for Vision. Eur J Dent 14(1):186–188. 10.1055/s-0040-1705076 32168545 10.1055/s-0040-1705076 PMC7069761

[4] Sun LF, Wang CX, Cao ZY et al (2021) Evaluation of autofluorescence visualization system in the delineation of oral squamous cell carcinoma surgical margins. Photodiagnosis Photodyn Ther 36:102487. 10.1016/j.pdpdt.2021.102487 34411738 10.1016/j.pdpdt.2021.102487

[5] Miao X, Ma R, Li J et al (2024) Dynamic characterization of vascular response and treatment in oral traumatic ulcer in mice via photoacoustic imaging. Quant Imaging Med Surg 14(7):4333–4347. 10.21037/qims-24-123 39022262 10.21037/qims-24-123 PMC11250348

[6] Gorpas D, Davari P, Bec J et al (2018) Time-resolved fluorescence spectroscopy for the diagnosis of oral lichen planus. Clin Exp Dermatol 43(5):546–552. 10.1111/ced.13404 29436013 10.1111/ced.13404 PMC9008188

[7] Varghese SS, Sarojini SB, George GB et al (2015) Evaluation and comparison of the biopathology of collagen and inflammation in the extracellular matrix of oral epithelial dysplasias and inflammatory fibrous hyperplasia using picrosirius red stain and polarising microscopy: a preliminary study. J Cancer Prev 20(4):275–280. 10.15430/JCP.2015.20.4.275 26734590 10.15430/JCP.2015.20.4.275 PMC4699755

[8] Tiwari L, Kujan O, Farah CS (2020) Clinico-pathological correlation of optical fluorescence imaging in oral mucosal lesions. Oral Dis 26(6):1230–1239. 10.1111/odi.13334 32198955 10.1111/odi.13334

[9] Pavlova I, Williams M, El-Naggar A, Richards-Kortum R, Gillenwater A (2008) Understanding the biological basis of autofluorescence imaging for oral cancer detection: high-resolution fluorescence microscopy in viable tissue. Clin Cancer Res 14(8):2396–2404. 10.1158/1078-0432.CCR-07-1609 18413830 10.1158/1078-0432.CCR-07-1609 PMC2773159

[10] Balasubramaniam AM, Sriraman R, Sindhuja P et al (2015) Autofluorescence based diagnostic techniques for oral cancer. J Pharm Bioallied Sci 7(Suppl 2):S374–S377. 10.4103/0975-7406.163456 26538880 10.4103/0975-7406.163456 PMC4606622

[11] Skala MC, Riching KM, Bird DK et al (2007) In vivo multiphoton fluorescence lifetime imaging of protein-bound and free nicotinamide adenine dinucleotide in normal and precancerous epithelia. J Biomed Opt 12(2):024014. 10.1117/1.2717503 17477729 10.1117/1.2717503 PMC2743958

[12] Sun Y, Phipps J, Elson DS et al (2009) Fluorescence lifetime imaging microscopy: in vivo application to diagnosis of oral carcinoma. Opt Lett 34(13):2081–2083. 10.1364/ol.34.002081 19572006 10.1364/ol.34.002081 PMC4083182

[13] Romano A, Di Stasio D, Petruzzi M et al (2021) Noninvasive imaging methods to improve the diagnosis of oral carcinoma and its precursors: state of the art and proposal of a three-step diagnostic process. Cancers (Basel) 13(12):2864. 10.3390/cancers13122864 34201237 10.3390/cancers13122864 PMC8228647

[14] Lane PM, Gilhuly T, Whitehead P et al (2006) Simple device for the direct visualization of oral-cavity tissue fluorescence. J Biomed Opt 11(2):024006. 10.1117/1.2193157 16674196 10.1117/1.2193157

[15] Andrade SA, Pratavieira S, Bagnato VS, Varotti FP (2021) Use of wide-field optical fluorescence for visualization of oral biofilm in a patient with peri-implant mucositis: a new approach. Einstein (Sao Paulo) 19:eRC5638. 10.31744/einstein_journal/2021RC5638 34037088 10.31744/einstein_journal/2021RC5638 PMC8121375

